# Dietary strategies and nutritional supplements in the management of heart failure: a systematic review

**DOI:** 10.3389/fnut.2024.1428010

**Published:** 2024-10-11

**Authors:** Xinyi Yu, Qilan Chen, Inmaculada Xu Lou

**Affiliations:** Department of Cardiology, Hangzhou TCM Hospital of Zhejiang Chinese Medical University (Hangzhou Hospital of Traditional Chinese Medicine), Hangzhou, China

**Keywords:** heart failure, diet, endothelial dysfunction, nutrition, supplements

## Abstract

**Background and objective:**

Heart failure (HF) is a syndrome of increased intracardiac pressure or decreased cardiac output. There is a lack of conclusive evidence to recommend the regular use of any dietary supplement in patients with HF. However, certain studies have shown nutritional interventions to be beneficial for patients with HF. Therefore, the purpose of this systematic review was to understand and map the updates of dietary interventions and nutritional supplementation measures related to patients with HF over the past 5 years.

**Study design:**

A systematic review.

**Methods:**

The PubMed, Web of Science, Scopus, and Cochrane Library databases were searched for randomized clinical trials on the association between dietary interventions and nutritional supplements and HF published between 2018 and 2023. A total of 1755 documents were retrieved, of which 19 were finalized for inclusion.

**Results:**

The findings suggest that individualized nutritional support reduces mortality and risk of major cardiovascular events in chronic heart failure inpatients at high nutritional risk. The Mediterranean diet improves functionality, quality of life, and cardiac function. Additionally, supplementation with thiamine, ubiquinol, D-ribose, and L-arginine enhances left ventricular ejection fraction. Probiotic yogurt may effectively improve the inflammatory and antioxidative status of chronic heart failure. Whey protein and melatonin have a positive effect on improving endothelial function in HF patients.

**Conclusion:**

Certain dietary interventions and nutritional supplements may provide some benefit to patients with HF. However, there is no relevant definitive evidence on the impact of nutritional interventions on the prognosis of HF, and more high-quality clinical trials are needed for further in-depth studies.

**Systematic review registration:**

Identifier, CRD42024510847

## Introduction

Heart failure (HF) is a syndrome characterized by structural or functional abnormalities of the heart, leading to increased intracardiac pressure or reduced cardiac output (1). Presently, an estimated 64.3 million individuals worldwide are afflicted by this condition (2). Its elevated rates of hospitalization and mortality place a considerable economic strain on public healthcare systems, rendering it a grave clinical and public health concern (3). Clinical management strategies for HF patients presently emphasize optimizing neurohormonal blockade and regulating volume status, often neglecting nutritional status assessment (4).

Heart failure exhibits significant gender differences, rooted in the inherent biological variations between men and women in cardiovascular system structure and function (5, 6). These differences influence risk factors, pathophysiology, clinical presentation, diagnosis, and treatment responses (6). Emerging evidence highlights notable gender differences in how nutritional status affects heart failure outcomes. Kwaśny et al. (7) found that underweight (body mass index, BMI < 18.5) and malnutrition (nutritional risk score, NRS ≥ 3) significantly increased the risk of in-hospital mortality by 15.98 times and 4.69 times, respectively, in male heart failure patients, but not in female patients. This finding suggests that gender-specific nutritional assessments may play a crucial role in optimizing individual heart failure treatment. However, the mechanisms behind these gender-specific nutritional effects are not yet fully understood (8).

The adult heart is a “metabolic omnivore,” using various substrates like amino acids, ketone bodies, fatty acids (FAs), and glucose to generate Adenosine triphosphate (ATP) (9). Around 95% of the heart’s ATP is produced through mitochondrial oxidative phosphorylation, with the remaining 5% coming mainly from glycolysis (10). Of the ATP generated by mitochondria, 40–60% is derived from FAs oxidation (11). Remarkably, the heart is estimated to hydrolyze more than 6 kilograms of ATP daily. In heart failure, energy metabolism undergoes significant changes. There is a decrease in FA metabolism and an increase in glucose utilization and glycolytic activity (11). This metabolic shift helps protect cardiomyocytes from oxidative stress and cellular damage (12, 13). However, this transition results in an insufficient ATP supply and abnormal accumulation and transport of metabolites (14). The increase in glycolytic activity also leads to higher lactate production and proton levels, which reduce cardiac efficiency and impair heart function (15). It can be assumed that combining metabolic therapies with optimized nutrition could synergistically enhance energy production and improve cardiac function in heart failure patients (16).

Nutritional therapy for HF patients should prioritize the individual’s BMI. Although this approach is subject to debate, total caloric intake and the macronutrient composition of the diet remain essential (17, 18). Studies indicate that patients with high BMI and metabolic alterations benefit significantly from caloric restriction (−30% kcal), combined with low carbohydrate and fat intake. For patients with high BMI but normal metabolism, or those with normal BMI, moderate caloric restriction (−15% kcal) and reduced carbohydrate intake are recommended. Conversely, low BMI patients require increased caloric intake (+30% kcal) with higher carbohydrate and fat consumption (13). However, due to intestinal edema, patients with right heart failure are prone to malabsorption and increased gut permeability. Insufficient energy intake may result in compromised nutritional status in heart failure patients, necessitating hospitalization for intervention. Additionally, chronic heart failure patients may suffer from sarcopenia due to fatigue and shortness of breath, resulting in decreased daily activity levels aggravated by malnutrition. Decreased skeletal muscle mass can compromise skeletal muscle function, worsening malnutrition as heart failure progresses and cardiac volume load increases, potentially culminating in cardiac cachexia (19).

Obesity (BMI ≥ 30 kg/m^2^) is a well-established independent risk factor for HF (20, 21). However, the “obesity paradox” in chronic HF suggests that higher BMI is associated with lower mortality rates (22). Nonetheless, a meta-analysis indicates that the obesity paradox should no longer be considered a significant prognostic factor (23, 24). Studies show that after comprehensive adjustment for multiple factors, patients with heart failure with reduced ejection fraction (HFrEF) in the PARADIGM-HF study no longer exhibit a BMI-related “obesity-survival paradox” (23). Additionally, Czapla et al. (25) reported that an increase in BMI is associated with reduced in-hospital mortality in men, whereas the opposite is observed in women. Therefore, the “obesity paradox” should be interpreted with caution. Early studies on the “obesity paradox” overlooked other prognostic variables such as sex, age, nutritional status, and treatment strategies (26, 27). Furthermore, most studies have used BMI alone to assess obesity, but BMI does not account for the composition of muscle, fat, and bone, nor does it reflect the distribution of fat within the body (24, 28). The distribution of visceral adipose tissue and muscle strength significantly impacts the prognosis of cardiovascular disease patients (29, 30). Therefore, it is necessary to go beyond BMI by considering socioeconomic status, medication adherence, and body composition analysis for a more comprehensive assessment of HF patients’ health status, thereby optimizing management strategies (25). Currently, large-scale clinical trials addressing cachexia or sarcopenia in HF patients are lacking, as are comprehensive dietary guidelines. Research results suggest that the Mediterranean diet has a protective effect on the incidence of heart failure or deterioration of cardiac function parameters in patients with previous cardiovascular disease, and that adherence to the Mediterranean or ‘Dietary Approaches to Stop Hypertension’ (DASH) diet may benefit primary prevention of HF (31). In addition, increasing protein and calorie intake, such as adhering to the PROT-AGE protein target (1.0–1.2 g/kg body weight/day), may be beneficial (32). Micronutrients also play a crucial role in human growth, metabolism, and immune system function (33), thus supplementing them can improve quality of life and potentially survival rates (34). Despite the widespread use of various nutritional supplements among HF patients, evidence in this area remains limited (35). Therefore, this review aims to identify and outline recent dietary interventions and nutritional supplementation measures for HF patients in the past 5 years.

## Methods

### Search strategy

From December 2023 to January 2024, a systematic search was performed across prominent databases including Pubmed, Web of Science, Scopus, and the Cochrane Library. The search spanned the preceding 5 years, with a primary focus on randomized controlled trials. This inquiry aims to elucidate the influence of dietary interventions and nutritional supplements on outcomes associated with heart failure. The search formula employed was as stated below: (“Heart Failure” OR “Cardiac Failure” OR “Heart Decompensation” OR “Myocardial Failure” OR “Congestive Heart Failure” OR “Left ventricular dysfunction” OR “Cardiac dysfunction” OR “BNP” OR “Ejection fraction” OR “CHF” OR “HFrEF” OR “HFpEF”)AND (“Nutrition” OR “Dietary” OR “Nutrient” OR “Diet” OR “Macronutrient” OR “Micronutrient” OR “Malnutrition” OR “Nutritional” OR “Antioxidant” OR “Eat” OR “Food” OR “Caloric restriction” OR “Mediterranean diet” OR “MedDiet” OR “Vitamin” OR “Mineral” OR “Polyunsaturated fatty acids” OR “PUFA” OR “omega-3” OR “omega-6” OR “coenzyme Q10” OR “CoQ10” OR “Cachexia” OR “Supplement” OR “Protein” OR “Energy” OR “Salt” OR “Water” OR “Cholesterol” OR “Liquid” OR “Thiamine” OR “Glucose” OR “Potassium” OR “Phosphorus” OR “Magnesium” OR “Creatine” OR “Carnitine” OR “Ubiquinone” OR “Taurine” OR “Fatty acid” OR “DASH” OR “Carbohydrate” OR “Fat” OR “Iron” OR “Fasting” OR “Calcium”).

### Inclusion criteria

The inclusion criteria for this search were as follows: (1) original articles investigating the efficacy of dietary interventions or oral nutritional supplements on outcomes related to HF; (2) published between 2018 and 2023; (3) articles published in English; (4) randomized controlled trials; (5) trials conducted in populations aged 18 and above, including both males and/or females; (6) full-text availability; (7) methodological quality scored greater than 3 points on the JADAD scale (36).

### Exclusion criteria

Exclusion criteria included: (1) studies involving non-human subjects; (2) studies not pertinent to the review topic; (3) studies lacking adequate description of dietary interventions and their impact on cardiovascular or other outcomes.

Two investigators were tasked with conducting the document search and screening process. Any discrepancies were resolved by a third researcher. This study has been registered in the International Prospective Register of Systematic Reviews (PROSPERO) under the registration code [CRD42024510847].

## Results

### Study characteristics

**Figure 1:**
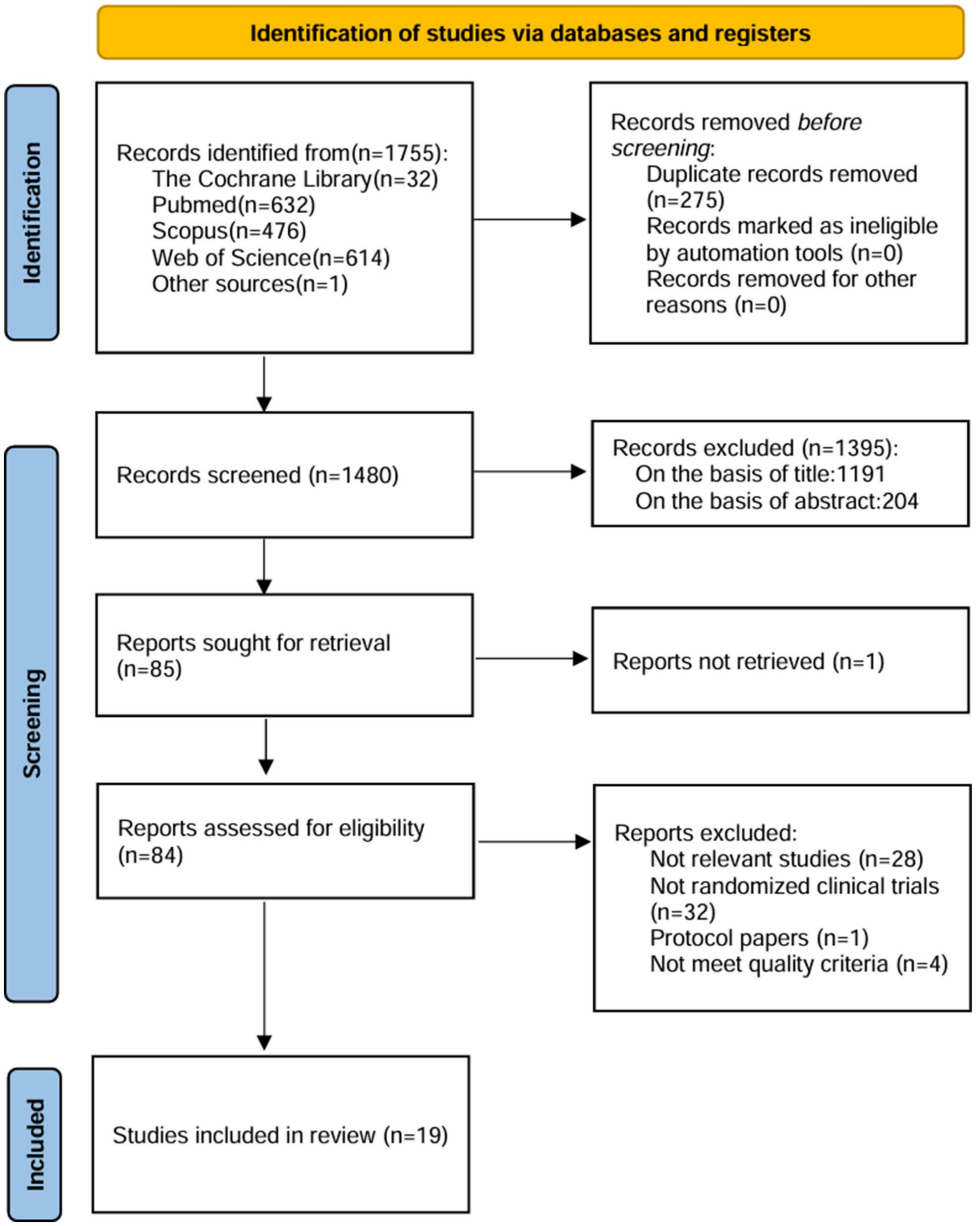
PRISMA flow diagram.

Initially, a total of 1755 documents were screened, resulting in the inclusion of 19 studies for analysis. The selection process of these studies, delineated in Figure 1, is exhaustively elucidated within this review. Key characteristics of the included studies are meticulously outlined in Table 1. It is noteworthy that the 19 randomized controlled trials (RCTs) included in the study all adhered to rigorous epidemiological designs.

**Table 1:** Main characteristics of the studies included in this review.

**Authors**	Sample size (N), Mean age, Sex (F/M)	Cardiovascular parameters	Study design	Intervention	Duration	Clinical Outcomes
Lorenzo et al. (42)	*N* = 33 (I:15, C:10) I:64.5, C:68.2 6/19	CVC	RCT	30 g WP	12 weeks	WP enhanced peak CVC during skin iontophoresis of SNP (*p* = 0.04) and Ach (*p* = 0.03) versus placebo.
Ezekowitz et al. (46)	*N* = 806 (I:397, C:409) I:66, C:67 268/538	CV-related hospitalization, CV-related emergency department visit, all-cause mortality, KCCQ Scores, NYHA functional class, 6MWT	RCT	Low sodium	12 months	No difference in the primary outcome (a composite of CV-related hospitalization, CV-related emergency department visit, and all-cause mortality, *p* = 0.53). Significant improvements in KCCQ and NYHA functional class (*p* = 0.0061). No change in 6MWT (*p* = 0.41).
Fabricio et al. (47)	*N* = 44 (I:22, C:22) I:59.5, C:56.4 59.1% (male)	Serum sodium value, incidence of hyponatremia, blood pressure (SBP, DBP, MAP), heart rate, NT-ProBNP, mean hospitalization duration, 30-Day readmission rate	RCT	Low sodium	7 days	LS achieved similar decongestion compared to NS. NS led to lower NT-proBNP levels (*p* = 0.04), higher mean blood pressure (*p* = 0.03), and lower heart rate (*p* = 0.02). LS had lower serum sodium levels (p = 0.04) and showed a higher incidence of hyponatremia (22%). Mean hospitalization duration was shorter in the NS group (*p* = 0.02). No significant difference in 30-day readmission rates between groups (*p* = 1.0).
Montgomery et al. (48)	*N* = 65 (I:34, C:31) I:70, C:70 24/41	Serum creatinine level, weight, blood pressure, serum sodium level, serum BUN level	RCT	6 g/d NaCl capsules	96 h	No statistically significant difference in treatment efficacy (change in serum creatinine and weight, *p* = 0.33) and safety endpoints. Significant differences in changes in serum sodium (*p* < 0.001) and serum BUN (*p* = 0.025).
Hassanzadeh-Makoui et al. (37)	*N* = 82 (I:41, C:41) I:61.68, C:62.12 39/43	LVEF, EDV, EDWT, NYHA functional class	RCT	50,000 IU/week vitamin D	8 weeks	Improvement in EF and EDV in the vitamin D group (*p* < 0.001). Increase in serum albumin (*p* = 0.036) and vitamin D levels (*p* < 0.001) in the vitamin D group. Significant improvement in NYHA class (*p* < 0.001).
Zittermann et al. (38)	*N* = 161 (I:80, C:81) I:55.5, C:54.0 30/131	Total-cholesterol, HDL-cholesterol, LDL-cholesterol, triglycerides, total-cholesterol/HDL-cholesterol ratio, LDL-cholesterol/HDL-cholesterol ratio, Fetuin A, dp-ucMGP	RCT	4,000 IU/d Vitamin D	3 years	No significant differences in lipid parameters or VC markers between groups (*p* values: 0.395–0.939). No sex-specific vitamin D effects (*p* = 0.828). No significant treatment effect on lipid parameters or VC markers in subgroup analyses in patients with 25OHD concentrations <30 nmol/L, nonusers of lipid-lowering drugs, or diabetic patients (p values: 0.245–0.998).
Zittermann et al. (39)	*N* = 133 (I:71, C:62) I:55.0, C:51.1 0/133	Calcium, 25OHD, 1,25(OH)_2_D, iPTH, TT, SHBG, fT, BAT	RCT	4,000 IU/d Vitamin D	3 years	Adjusted between-group differences significant for plasma calcium (*p* = 0.003), 25OHD (*p* < 0.001), and 1,25(OH)2D (*p* = 0.003); not for iPTH (*p* = 0.182). No between-group differences in TT (*p* = 0.612), SHBG (*p* = 0.393), fT (*p* = 0.861), or BAT (*p* = 0.960).
Hearon et al. (55)	*N* = 56 (HIIT + n-3 FA:13, HIIT + Pbo:16, n-3 FA:14, Pbo:13) HIIT + n-3 FA:50, HIIT + Pbo:50, n-3 FA:47, Pbo: 49 32/24	Peak oxygen uptake (VO₂), cardiac output, LV mass and volume, epicardial LV borders, epicardial LV borders, arterial stiffness, blood pressure	RCT	HIIT, 1.6 g/d n-3 FA	1 year	No independent or interaction effect of n-3 FA supplementation. No detectible effect of HIIT on visceral fat or myocardial triglyceride content despite a reduction in total adiposity (*p* = 0.018). HIIT: ~24% improved exercise capacity (*p* < 0.0001), increased LV mass (*p* < 0.001) and volume (*p* < 0.001), reduced augmentation index (*p* = 0.009). No effect of either intervention on markers of arterial stiffness.
Hoseini et al. (40)	*N* = 92 (I:46, C:46) I:63.5, C:58.5 12/80	LVEF, LVEDD, LVESD, NT-ProBNP, All-cause mortality, hospitalization for HF exacerbation, MLHFQ, hs-CRP, total-cholesterol, HDL-cholesterol, LDL-cholesterol, triglycerides, NYHA	RCT	10 mg/d melatonin	24 weeks	Serum NT-Pro BNP decreased significantly in the melatonin group compared to placebo (*p* = 0.044). Composite clinical outcome^a^ (*p* = 0.017), MLHFQ score (*p* = 0.037), and NYHA class (*p* = 0.015) improved in the melatonin group compared to placebo. No significant difference in echocardiographic parameters. No mortality during the study.
Hoseini et al. (41)	*N* = 92 (I:46, C:46) I:62.7, C:59.1 12/80	FMD, blood pressure, lipid profile, NT-ProBNP, MDA, TAC	RCT	10 mg/d melatonin	24 weeks	Improvement in FMD in melatonin group compared to placebo (*p* = 0.027). No significant difference in systolic/diastolic blood pressure, serum total antioxidant capacity, and serum MDA. Improvement in FMD and MDA in nondiabetic patients in the melatonin group compared to placebo. No significant difference in FMD and MDA between groups in diabetic patients.
Keith et al. (43)	*N* = 69 (I:35, C:34) I:64, C:63 11/58	LVEF, MLHFQ, 6MWT, NT-ProBNP, Troponin I	RCT	200 mg/d thiamin	6 months	Improvement in erythrocyte TPP (*p* = 0.02) and urine thiamin (*p* < 0.001) in thiamin group compared to placebo. LVEF significantly higher in placebo compared to thiamin group (*p* = 0.047). No significant differences in MLHFQ, 6MWT, Troponin I and NT-proBNP.
Pierce et al. (56)	*N* = 139 (UC:35, Pbo:38, DR:33, UC + DR:33) 68.7 (overall) 78/61	KCCQ, EF, E/e’ Ratio, BNP, 6MWT	RCT	600 mg/d Ubiquinol capsules, 15 g/d D-Ribose Powder	12 weeks	Improvement in KCCQ clinical summary score, vigor score, and EF in ubiquinol and/or D-ribose group compared to placebo. Reduction in BNP and lactate/ATP ratio in ubiquinol and/or D-ribose group compared to placebo. No significant changes in the septal E/e’ ratio or the 6MWT in each group.
Pourrajab et al. (49)	*N* = 78 (I:39, C:39) I:55.59, C:53.87 23/55	NT-ProBNP, oxLDL, ApoB100, PTX3	RCT	300 mL/d probiotic yogurt	10 weeks	Reduction significantly in oxLDL in probiotic yogurt compared to ordinary yogurt (adjusted *p* = 0.010), but not significant for PTX3 (adjusted *p* = 0.236). No significant effects on the NT-proBNP (adjusted *p* = 0.306) and ApoB100 levels (adjusted *p* = 0.280).
Pourrajab et al. (50)	*N* = 78 (I:39, C:39) I:55.59, C:53.87 23/55	sTWEAK, sCD163, ADMA, LCAT, BUN	RCT	300 mL/d probiotic yogurt	10 weeks	Improvement in sTWEAK was significant in the probiotic yogurt group compared to the ordinary yogurt group (adjusted *p* = 0.038). No significant differences on the sCD163、ADMA、LCAT、BUN.
Sabbaghzadegan et al. (52)	*N* = 42 (I:21, C:21) I:55.9, C:57.1 16/26	MLHFQ, 6MWT, NYHA functional class	RCT	300 mg/d AVG capsule	8 weeks	Reduction in MLHFQ (*p* < 0.001) and NYHA (*p* = 0.004) functional class in the AVG compared to placebo. The change of 6MWT in the AVG group was more advanced but not statistically significant (*p* = 0.353). ISI (*p* < 0.001), STOP-BANG (*p* = 0.01) and PSQI (*p* < 0.001) improved in the AVG group compared to placebo. Fewer adverse events reported in the AVG group (*p* = 0.047)
Salmani et al. (44)	*N* = 42 (I:21, C:21) I:55.9, C:57.1 16/26	SBP, DBP, MAP, LVDd, LVDs, LVEF, left ventricular function, systolic function, diastolic dysfunction, 6MWT, MLHFQ	RCT	3 g/d L-arginine	10 weeks	Improvement in EF (*p* = 0.037), left ventricular function (*p* = 0.043), diastolic dysfunction (*p* = 0.01), and LVDd (*p* = 0.065), physical (*p* = 0.002) and total scores (*p* = 0.011) of QoL in the L-arginine group compared to placebo group. Significant improvements in DBP, MAP, LVDd, EF, left ventricular function, diastolic dysfunction, and QoL scores compared to baseline (all *p* < 0.05), but not in the placebo group.
Samuel et al. (45)	*N* = 44 (I:23, C:21) I:56.6, C:55.8 13/31	LVEF, lateral e’, lateral E/e’, E/A, LVEDD, LV mass index, NT-proBNP	RCT	300 mg/d CoQ10	4 months	No significantly affect on NT-proBNP levels. No significant effect on indices of diastolic function (*p* = 0.561). No significant difference in LVEF between intragroup changes.
Herrera-Martínez et al. (53)	*N* = 38 (I:19, C:19) I:65, C:72 27/11	LVEF, NT-proBNP, Total cholesterol, HDL cholesterol, LDL cholesterol, triglycerides, HF hospitalizations, Mortality	RCT	MedDiet, Standard hypercaloric, hyperproteic oral supplement	24 weeks	LVEF increased in the whole cohort (*p* < 0.01); higher in the intervention group (*p* < 0.05). Serum values of NT-proBNP significantly decreased in the whole cohort (*p* < 0.01), especially in the intervention group (*p* = 0.02). No significant association with mortality or new hospital admissions for nutritional support, baseline LVEF, NT-proBNP, body composition parameters, or functionality tests.
Hersberger et al. (54)	*N* = 645 (I:321, C:324) I:78.7, C:79.0 314/331	All-cause mortality day 30, all-cause mortality day 180, MACE (myocardial infarction, stroke, death within 30 days), Major Complications (MACE, acute renal failure, infection requiring antibiotic treatment within 30 days), Index Hospital LOS, Nonelective Hospital Readmission, Need for ICU Admission	RCT	Individualized nutritional support	30 days	Significant long-term reduction in mortality at 180 days in the intervention group (26.5% vs. 31.5%; p = 0.047). High nutritional risk (NRS score > 4) increased 180-day mortality by 65% compared to moderate risk (NRS score 3–4; 24.7% vs. 38.4%, *p* = 0.001). Lower mortality in the intervention group at 30 days (8.4% vs. 14.8%, p = 0.002). Greater benefit from nutritional support in high nutritional risk (NRS > 4) compared to moderate risk. Significantly lower risk of MACE in the intervention group within 30 days (17.4% vs. 26.9%, *p* = 0.001). No significant difference in ICU admission or LOS.

1,25 (OH)_2;_ D, 1,25-dihydroxyvitamin D; 25OHD, 25-hydroxyvitamin D; 6MWT, 6-min walk distance; Ach, acetylcholine; ADMA, asymmetric dimethylarginine; ApoB100, apolipoprotein B100; ATP, adenosine triphosphate; AVG, Aloe vera gel; BAT, bioactive testosterone; BUN, blood urea nitrogen; C, control; CoQ10, Coenzyme Q10; CV, cardiovascular; CVC, cutaneous vascular conductance; DBP, diastolic blood pressure; dp-ucMGP, non-phosphorylated undercarboxylated matrix gla protein; DR, D-Ribose Powder group; EDV, end-diastolic volume; EDWT, end-diastolic wall thickness; EF, ejection fraction; E/A, Ratio of early to late diastolic velocities; E/e’, Ratio of mitral peak velocity of early filling (E) to early diastolic mitral annular tissue velocity (e’); F, female; FMD, flow-mediated dilatation; fT, free testosterone; HDL, high density lipoprotein; HF, heart failure; HIIT, high-intensity interval training; hs-CRP, high-sensitivity C-reactive protein; I, intervention; ICU, intensive care unit; iPTH, intact parathyroid hormone; KCCQ, Kansas City Cardiomyopathy Questionnaire; LCAT, lecithin cholesterol acyltransferase; LDL, low density lipoprotein; LOS, length of stay; LS, low sodium diet; LV, left ventricular; LVDd, left ventricular dimension during diastole; LVDs, left ventricular dimension during systole; LVEDD, left ventricular end-diastolic diameter; LVEF, left ventricular ejection fraction; LVESD, left ventricular end-systolic diameter; M, male; MACE, Major adverse cardiovascular events; MAP, mean arterial pressure; MDA, malondialdehyde; MedDiet, Mediterranean Diet; MLHFQ, Minnesota Living with Heart Failure Questionnaire; n-3 FA, omega-3 fatty acid; NRS, Nutritional Risk Screening 2002; NS, normal sodium diet; NT-Pro BNP, N-terminal pro-B-type natriuretic peptide; NYHA, New York Heart Association; oxLDL, oxidized low density lipoprotein; Pbo, Placebo; PSQI, Pittsburgh Sleep Quality Index; PTX3, pentraxin3; QoL, quality of life; RCT, randomized clinical trial; SBP, systolic blood pressure; sCD163, soluble cluster of differentiation 163; SHBG, sex hormone-binding globulin; SNP, sodium nitroprusside; sTWEAK, soluble tumor necrosis factor-like weak inducer of apoptosis; TAC, total antioxidant capacity; TPP, thiamin pyrophosphate; TT, total testosterone; UC, Ubiquinol capsules group; VC, vascular calcification; WP, whey protein; ^a^Composite clinical outcome consisted of all-cause mortality, hospitalization for HF exacerbation, and changes in quality of life measured by MLHFQ.

### Main results from the experimental studies

The nutritional interventions evaluated for HF in the studies included nutritional supplements, dietary strategies, and combined therapies.

### Nutritional supplements

Hassanzadeh-Makoui et al. (37) observed that administering 50,000 IU of vitamin D per week for 8 weeks resulted in significant improvements in primary outcomes, such as ejection fraction (EF) and end-diastolic volume, in the treatment group compared to the placebo group (*p* < 0.001). Secondary outcomes, including serum albumin (*p* = 0.036) and vitamin D levels (*p* < 0.001), also increased significantly. Additionally, the treatment group exhibited more pronounced improvements in New York Heart Association (NYHA) class compared to the placebo group (*p* < 0.001). Zittermann et al. (38, 39) found that oral administration of 4,000 IU of Vitamin D per day for 3 years did not significantly affect lipid parameters and vascular calcification (VC) markers in patients with advanced HF (*p* values: 0.395–0.939). Additionally, no gender-specific effects of vitamin D were observed. Furthermore, in a pre-specified secondary analysis of the EVITA (effect of vitamin D on mortality in heart failure) randomized controlled trial, they found that continuous supplementation of 4,000 IU of vitamin D3 per day for 3 years did not prevent the decline in hormone indices in male patients with advanced HF and low 25-hydroxyvitamin D (25OHD) concentration.

Hoseini et al. (40, 41) observed that supplementation with 10 mg of melatonin daily for 24 weeks led to a significant decrease in serum N-terminal pro-B-type natriuretic peptide (NT-proBNP) (*p* = 0.044), as well as significant improvements in composite clinical outcome (*p* = 0.017), quality of life (*p* = 0.037), and NYHA classification (*p* = 0.015). However, melatonin did not affect the echocardiographic parameters in patients. Furthermore, they noted that flow-mediated dilatation (FMD) was significantly better after melatonin treatment compared to the placebo group (*p* = 0.027). Subgroup analysis revealed that in non-diabetic patients, both FMD and malondialdehyde (MDA) significantly improved in the melatonin group, while there was no difference between the two groups in diabetic patients.

Lorenzo et al. (42) reported that supplementing with whey protein (WP) for 12 weeks in patients with NYHA classes I/II HF led to increased endothelium-dependent (*p* = 0.03) and endothelium-independent vasodilation (*p* = 0.04). Keith et al. (43) discovered that administering 200 mg/day of thiamin for 6 months to HF patients with left ventricular ejection fraction (LVEF) < 40% resulted in a significant increase in thiamin concentrations in both blood and urine (*p* = 0.02 and < 0.001, respectively). However, oral thiamin supplementation for 6 months did not improve LVEF. Additionally, thiamin supplementation did not improve Minnesota Living with Heart Failure Questionnaire (MLHFQ) or 6-min walk distance (6MWT), nor did it reduce the concentrations of NT-proBNP or Troponin I. Pierce et al. (37) observed significant improvements in HFpEF patients treated with ubiquinol and/or D-ribose for 12 weeks, with Kansas City Cardiomyopathy Questionnaire (KCCQ) clinical summary score increasing from 17.30 to 25.82 points, vigor score from 7.65 to 8.15 points, and EF from 72.02 to 47.51, while reducing B-type natriuretic peptide from −72.02 to −47.51 and lactate/ATP from −4.32 to −3.35*10^−4^. No significant increase was observed in E/e’ ratio or 6-min walk test. Moreover, they observed that adding D-ribose to ubiquinol did not have a synergistic effect. Salmani et al. (44) discovered that administering 3 g/day of L-arginine for 10 weeks resulted in significant improvements compared to the placebo group in EF (*p* = 0.037), left ventricular function (*p* = 0.043), diastolic dysfunction (*p* = 0.01), as well as physical (*p* = 0.002) and total (*p* = 0.011) quality of life scores, with a slight improvement in left ventricular dimension during diastole (LVDd) (*p* = 0.065). However, Samuel et al. (45) discovered that supplementing with 300 mg of Coenzyme Q10 (CoQ10) per day for 4 months had no significant effect on echocardiographic diastolic function indices (difference in the lateral E/e’ ratio: *p* = 0.561) and serum NT-proBNP levels (*p* = 0.163).

### Dietary strategies

Ezekowitz et al. (46) conducted a 12-month daily low-sodium diet intervention of less than 1,500 milligrams in patients with NYHA class II/III CHF. The results indicated no significant difference in the primary outcome, encompassing all-cause mortality (*p* = 0.32), hospitalization due to cardiovascular reasons (*p* = 0.36), and visits to the emergency department related to cardiovascular issues (*p* = 0.60). However, moderate improvements in quality of life, as assessed by KCCQ and NYHA functional class, were observed, despite the lack of statistical significance in the 6MWT between the two groups (*p* = 0.41). Fabricio et al. (47) compared a 7-day intervention of normal sodium diet (7 g/day) with low sodium diet (3 g/day of dietary sodium chloride) to evaluate the protective effects of a normal sodium diet combined with fluid restriction on sodiumemia and blood pressure in patients with acute decompensated HF. They found that compared to normal sodium diet, low sodium diet had no advantage in alleviating congestion, but normal sodium diet led to lower NT-proBNP levels (*p* = 0.04), higher mean blood pressure (*p* = 0.03), and lower heart rate (*p* = 0.02). Furthermore, at the end of the intervention, 22% of patients in the low sodium group developed hyponatremia, primarily due to depletion. During intravenous diuresis in AHF patients, Montgomery et al. (48) administered oral doses of 2 g NaCl three times a day for 96 h and observed no statistically significant differences in serum creatinine and weight changes between the NaCl and placebo groups (*p* = 0.33).

Pourrajab et al. (49, 50) observed that the group consuming 300 mL of probiotic yogurt per day showed a significant decrease in oxidized low density lipoprotein (oxLDL) levels (adjusted *p* = 0.010) and an increase in soluble tumor necrosis factor-like weak inducer of apoptosis (sTWEAK) levels at week 10 compared to the regular yogurt group. Additionally, both probiotic yogurt and regular yogurt reduced Pentraxin-3 (PTX3) levels, although there was no significant difference between the two groups at the end of the 10-week intervention period (adjusted *p* = 0.236). PTX-3, also known as “long pentraxin,” is part of the evolutionarily conserved pentraxin family (51). Sabbaghzadegan et al. (52) indicated that administering oral AVG (*Aloe vera* gel) capsules of 150 mg twice daily for 8 weeks resulted in significant reductions in MLHFQ (*p* < 0.001) and NYHA functional class (*p* = 0.004), with fewer reported adverse events (*p* = 0.047), compared to the placebo group. Additionally, the severity of insomnia, obstructive sleep apnea, and sleep quality improved in the AVG group compared to the placebo group. However, despite more significant changes in the 6MWT in the AVG group, it was not statistically significant (*p* = 0.353).

In the realm of dietary modification research, Herrera-Martínez et al. (53) conducted an intervention study targeting HF patients with LVEF < 50%. They prescribed a Mediterranean diet (as the control group) along with two hypercaloric, hyperproteic oral supplements (OSs) (as the intervention group) for 24 weeks, supplemented with varying doses of calcifediol to achieve serum 25 OH vitamin D levels > 30 ng/dL. The findings revealed noteworthy enhancements in weight and BMI across the entire cohort (*p* = 0.02); however, no statistically significant differences were observed in abdominal, arm, or calf perimeters. In the group adhering solely to the Mediterranean diet in the nutritional intervention, improvements were noted in functionality, quality of life, and cardiac function. Meanwhile, the combination of the Mediterranean diet with hypercaloric, hyperproteic OS led to increases in body cell mass, lean mass, and body mass (an absolute increase of 0.5, *p* = 0.03, 1.2 kg, *p* = 0.03, and 0.1 kg, *p* = 0.03, respectively), alongside improvements in biochemical nutritional parameters. Furthermore, the hypercaloric, hyperproteic OS group exhibited more substantial enhancements in functionality, quality of life, and LVEF, accompanied by a reduction in serum NT-proBNP levels (*p* = 0.02).

Hersberger et al. (54) observed that protocol-guided individualized nutritional support, aimed at achieving energy, protein, and micronutrient targets, significantly reduced 30-day mortality (8.4% vs. 14.8%; *p* = 0.002) and 180-day mortality (26.5% vs. 31.5%; *p* = 0.047) in chronic heart failure patients with a Nutritional Risk Screening 2002 score ≥ 3 (36% with acute decompensation). Additionally, personalized nutritional support significantly reduced the risk of major cardiovascular events within 30 days (17.4% vs. 26.9%; *p* = 0.001), although no differences were observed between the groups in terms of intensive care unit admission or length of stay. They noted that the severity of malnutrition was directly correlated with higher 180-day mortality (odds ratio per 1-point increase in Nutritional Risk Screening 2002 score: 1.65; *p* = 0.001). Moreover, patients with high nutritional risk (NRS > 4) benefited more from nutritional support compared to those with moderate risk. Individualized nutritional support also significantly improved quality of life, as measured by the European Quality of Life 5 Dimensions Index and Visual Analog Scale.

### Combined therapies

In prior research, dietary intervention has been frequently coupled with physical exercise. Hearon et al. (55) intervened with stage A HF patients, administering daily 1.6 g of omega-3 fatty acid (n-3FA) along with exercise training sessions 3–4 times per week, each lasting 30–60 min. Exercise prescriptions were tailored to individual participants’ heart rates, with the aim of gradually increasing both duration and intensity over the intervention period. The findings revealed that after 1 year, High-Intensity Interval Training (HIIT) resulted in a reduction of total fat (*p* = 0.018) but did not significantly impact visceral fat or myocardial triglyceride content. Furthermore, one year of HIIT led to an approximately 24% increase in exercise capacity (*p* < 0.0001), along with increases in left ventricular mass (*p* < 0.001) and volume (*p* < 0.001), while decreasing the augmentation index (*p* = 0.009). However, supplementation with n-3 FA showed no independent or interactive effects on body composition, visceral fat, or myocardial triglyceride content.

## Discussion

In this study, we conducted a systematic review of the effects of nutritional supplements and dietary interventions on patients with HF. We found that various nutritional supplements and dietary interventions may provide some degree of benefit in patients with HF, but there are also some limitations and challenges. Our study indicates that most nutritional supplements and dietary interventions can improve the quality of life and LVEF of HF patients (37, 40, 44, 46, 52, 56), but their effects on other echocardiographic parameters are limited (40, 45). This is also the case when the diet is adjusted to high-calorie, high-protein, and Mediterranean styles (53). Furthermore, only a few intervention measures have assessed key outcomes such as all-cause mortality, cardiovascular-related hospitalization rates, and the risk of HF occurrence in HF patients (40, 46). In addition, some studies have attempted combined therapy of dietary intervention with high-intensity interval training (55), which may positively impact cardiovascular health and exercise capacity in HF patients, but further research is needed to evaluate its long-term effects and safety.

The Mediterranean diet can improve cardiovascular health in the general population and is a promising intervention. In a prospective study of 37,308 Swedish males, Tektonidis et al. (57) discovered that strict adherence to the Mediterranean diet correlated with a 31% lower risk of HF occurrence. Our study suggests that nutritional intervention with the Mediterranean diet can enhance functionality, quality of life, and cardiac function in HF patients (53). The protective effects of the Mediterranean diet may stem from its suppression of inflammation and oxidative stress, as well as its prevention of myocardial remodeling. These mechanisms help reduce the deterioration of cardiac function and the occurrence of HF (58). The DASH diet, with its analogous main components to the Mediterranean diet, presents a notable avenue in HF treatment. Adhering to the DASH diet can lower blood pressure, thus preventing the occurrence of HF (59). According to observations by Levitan et al. (60, 61), women and men with the highest quartile of DASH index had a 37 and 22% lower risk of HF, respectively, compared to the lowest quartile. Furthermore, some evidence suggests that this diet can protect HF patients by preserving endothelial function, reducing oxidative stress, and exerting anti-inflammatory effects (62–64). It is noteworthy that since the blood pressure-lowering effect of the DASH diet remains irrespective of sodium intake restriction, it is highly likely that its antihypertensive effect is attributed more to the diet itself rather than sodium reduction (65). These findings emphasize the potential of the Mediterranean diet and the DASH diet as valuable dietary interventions in heart failure management. However, conducting nutritional intervention trials in the HF population often presents challenges. Firstly, HF is a complex clinical syndrome influenced by various factors, including etiology, anatomical, and physiological changes. Different types and severities of HF may have diverse effects on dietary patterns. Secondly, dietary patterns often consist of complex combinations of foods and nutrients, with their combined effects usually involving interactions of multiple mechanisms. However, most intervention studies have failed to adequately explain the causal relationship between food components and their active metabolites in the pathophysiology of HF.

Micronutrients serve as auxiliary agents in energy production, transfer, and in maintaining cardiac contractile function (66). Micronutrient deficiency is associated with the survival and quality of life of heart failure patients (67). According to the review results by Dragan et al. (68), micronutrients can improve the health outcomes of HF patients by improving symptoms, functional capacity, and LVEF, thereby enhancing their quality of life. Kkeveetil et al.’s (33) systematic review also found sufficient evidence to support large-scale trials of micronutrient supplementation in HF patients. The majority of viewpoints outlined in our review align with those presented in other articles within the field, suggesting a positive influence of micronutrients on the outcomes of HF patients. It is noteworthy that in our study, the benefits of micronutrients on the major clinical outcomes of HF primarily stem from the research by Hoseini et al. (40), whose trials demonstrate the positive effects of melatonin observed across comprehensive clinical outcomes, including mortality, hospitalization due to decompensated heart failure, and quality of life. However, during their study period, no mortality cases occurred, and hospitalization instances were relatively low. Consequently, the majority of melatonin’s clinical benefits might originate from its influence on the quality of life. This outcome may be related to the relatively small sample size in the study.

The impact of vitamin D supplementation on cardiovascular health remains a subject of debate (69). Vitamin D, a secosteroid, is essential for calcium homeostasis, bone health, and immune regulation (70). Deficiency in vitamin D can lead to Ca2+ overload in cardiac cells, resulting in impaired myocardial contraction and relaxation, as well as inflammation, fibrosis, and cardiomyocyte hypertrophy (71, 72). While animal studies highlight the significance of vitamin D for cardiovascular health, large Mendelian randomization studies and major intervention trials, such as VITAL and ViDA, have not substantiated its beneficial effects on cardiovascular outcomes (69). Our findings suggest that vitamin D supplementation may improve ejection fraction and end-diastolic volume (37). Observational studies also show a positive correlation between circulating 25OHD levels and male sex hormone concentrations (39); however, our study did not find an effect of vitamin D on gender or male hormone markers (38, 39). Given the potential influence of vitamin D on cardiovascular health and sex hormones, further research is warranted to thoroughly evaluate its role and mechanisms across different genders and individual characteristics.

Research on *aloe vera* as a nutritional factor in heart failure management has garnered significant attention. Sabbaghzadegan et al. (73) highlighted that *aloe vera* gel (AVG) can prevent and improve cardiovascular diseases through its antioxidant, anti-fibrotic, anti-inflammatory, metabolic regulation, and anti-atherosclerotic properties. Birari et al. (74) demonstrated that aloin, a component of *aloe vera*, enhances the activity of antioxidant enzymes like catalase and superoxide dismutase. It also helps maintain glutathione (GSH) levels in heart tissue, thereby aiding in the scavenging of free radicals and mitigating As2O3-induced cardiac toxicity. Aloin also significantly inhibited the increase of inflammatory cytokines (IL-6 and IL-1β) induced by As2O3. However, oral *aloe vera*, particularly aloe latex, may lower potassium levels, heightening the risk of digoxin toxicity (75). The concurrent use of diuretics and aloe latex can exacerbate potassium depletion, leading to arrhythmias, weakness, fatigue, muscle cramps, and constipation (76, 77). Therefore, it is crucial to consider the potential interactions between *aloe vera* and heart failure medications to comprehensively assess its benefits and risks. Due to these potential interactions, especially with medications like digoxin, there is currently insufficient evidence to support *aloe vera*’s use in heart failure treatment. Well-designed clinical trials are necessary to evaluate the efficacy and safety of *aloe vera* in managing heart failure.

Research on dietary sodium restriction as an intervention for heart failure is currently a prominent research area. Salt restriction aids in reducing blood pressure and alleviating complications arising from structural and functional abnormalities associated with HF (78). In a prospective observational study involving 36,019 participants followed for 7 years, adherence to the DASH (Dietary Approaches to Stop Hypertension) diet, known for its sodium restriction, was linked to a decreased occurrence of heart failure (60). Nevertheless, our findings indicate that neither limiting nor augmenting sodium intake demonstrated notable therapeutic effects (46–48). Importantly, heightened salt intake not only increases the fluid burden on the cardiovascular system but also indirectly contributes to HF through gut microbiota dysbiosis (79). Trimethylamine (TMA), generated by diverse gut microbiota, undergoes enzymatic conversion to Trimethylamine N-oxide (TMAO) by flavin-containing monooxygenase 3 (FMO3) in the liver (80). TMAO is thought to contribute to HF development by triggering inflammatory and oxidative stress pathways, resulting in myocardial hypertrophy, impaired cardiac mitochondrial function, diminished left ventricular pumping capacity, and fibrosis (81, 82). Consequently, modulating gut microbiota dysbiosis with probiotics in functional foods or capsules emerges as a crucial aspect for HF therapeutic objectives. Our study suggests that probiotic yogurt may effectively ameliorate the inflammatory and antioxidant profiles of CHF patients (49, 50). Future clinical investigations should explore various types and doses of probiotics, along with prolonged intervention durations.

Endothelial dysfunction (ED) is a pivotal pathological mechanism in HF, utilized for diagnosis, prognosis, and as a therapeutic target (83). Previous studies have demonstrated significant improvement in endothelial function in HFrEF patients with ubiquinol (84) and lycopene (85) supplementation. Moreover, our findings indicate that dietary supplementation with WP can enhance systemic microvascular function in HF patients (42). Nevertheless, there is insufficient evidence to substantiate the efficacy of orally administered probiotic yogurt in ameliorating endothelial dysfunction (50). Numerous studies have demonstrated the robust predictive ability of endothelial dysfunction assessed via FMD for adverse outcomes in CHF patients, including heart transplantation or cardiac death (86, 87). Additionally, enhanced FMD following optimal treatment may mitigate the risk of such adverse outcomes (88). It is noteworthy that endothelial dysfunction observed in HF patients primarily stems from elevated levels of superoxide radicals and other oxidant species at the vascular level (89). However, despite significant improvement in FMD among HFrEF patients with oral melatonin supplementation, statistical analysis did not reveal significant differences in oxidative stress markers such as total antioxidant capacity (TAC) and MDA (41). We hypothesize that the beneficial effects of melatonin on the endothelium may be mediated through alternative protective pathways or may be evident in other oxidative stress biomarkers not assessed in this study. Hence, future research should contemplate employing additional biomarkers to evaluate oxidative stress and endothelial function, interventions tailored for diverse populations, and assessing intervention effects across various doses and durations.

In addition to enhancing endothelial function, nutritional supplements can significantly influence HF management by targeting various pathological mechanisms. Research identifies three primary pathological pathways underlying HF symptoms: fluid retention, inflammatory response, and oxidative stress (90). Lennie et al. (91) designed a study to address these pathways by reducing dietary sodium intake and combining it with supplementation of Omega-3 fatty acids and lycopene. This approach is anticipated to alleviate HF symptoms, improve health-related quality of life, and extend the time to readmission or all-cause mortality. Lycopene mitigates oxidative stress by boosting cellular antioxidant capacity, protecting endothelial nitric oxide, reducing cell damage, and inhibiting inflammation pathways (92). Additionally, Omega-3 fatty acid supplementation addresses the inflammatory response by increasing Omega-3 fatty acid content in cell membranes and decreasing the production of inflammatory mediators, thereby effectively alleviating inflammation (93). However, our results indicate that the significant efficacy of Omega-3 fatty acids remains uncertain (55). This raises concerns about potential variability in responses to the same intervention across different populations. The VITAL study demonstrated that n-3 fatty acid supplementation significantly reduced the rate of hospitalization for HF recurrence among African Americans (94). This finding suggests that race and genetic background may significantly influence the effectiveness of nutritional interventions, underscoring the need for further research to determine the most suitable intervention strategies for specific subgroups.

This systematic review comprehensively analyzed dietary strategies and nutritional supplements in the management of HF. Our study identified potential benefits of nutritional interventions in managing HF patients, particularly for those at risk of or experiencing malnutrition. Our findings corroborate prior research suggesting that altering dietary strategies or supplement intake may enhance the prognosis of HF patients. Furthermore, during the literature search, numerous articles explored the association between ED and HF, as well as malnutrition and HF. Screening literature from the past 5 years suggests that these research topics have garnered substantial attention recently. However, we identified limitations in our study, notably the absence of randomized controlled trial data on the effects of various dietary interventions, including the DASH diet and ketogenic diet, on heart failure. Moreover, upon comparing the 18 documents included in this review, we observed heterogeneity among them, precluding a meta-analysis. Moreover, despite readmission rates and mortality being crucial clinical outcomes for HF patients, only two studies reported them, with the majority focusing on surrogate outcomes like quality of life, LVEF, and brain natriuretic peptide. Finally, this systematic review did not include a search for grey literature, and only English articles were considered, potentially overlooking relevant studies.

## Conclusion

In summary, individualized nutritional support may reduce all-cause mortality and the risk of major adverse cardiovascular events in patients with HF, especially those at high nutritional risk, emphasizing the importance of considering its potential long-term benefits. Interventions such as vitamin D, thiamine, L-arginine, the Mediterranean diet, and standard high-calorie, high-protein oral supplements have the potential to improve left ventricular ejection fraction. However, there is a lack of high-quality studies assessing the specific impact of these interventions on clinical outcomes. While a normal sodium diet, melatonin, ubiquinol, D-ribose, and the Mediterranean diet, along with standard high-calorie, high-protein oral supplements, have shown some benefits in improving natriuretic peptide levels, evidence on their effects on long-term clinical outcomes remains insufficient. Overall, although some nutritional interventions and supplements have demonstrated positive effects on HF-related biomarkers and symptoms, most studies lack randomized controlled trial data on HF-related clinical outcomes.

Overall, the current evidence quality is low, underscoring the need for further high-quality, long-term clinical trials. These studies are crucial to better understand the impact of these nutritional interventions on clinical outcomes in HF patients and to develop more effective dietary and nutritional strategies.

## Data Availability

The original contributions presented in the study are included in the article/supplementary material, further inquiries can be directed to the corresponding author.
